# 美国临床肿瘤学会Ⅳ期非小细胞肺癌化疗的临床实践指南更新

**DOI:** 10.3779/j.issn.1009-3419.2010.03.15

**Published:** 2010-03-20

**Authors:** Christopher G. AZZOLI, Sherman Baker JR, Sarah TEMIN, William PAO, Timothy ALIFF, Julie BRHMER, David H. JOHNSON, Janessa L. LASKIN, Gregory MASTERS, Daniel MILTON, Luke NORDQUIST, David G. PFISTER, Steven PIANTADOSI, Joan H. SCHILLER, Reily SMITH, Tomas J. SMITH, John R. STRWN, David TRENT, Giuseppe GIACCONE, 燕 丁, 娟 南, 谦 刘

**Affiliations:** 1 From the Memorial Sloan-Ketering Cancer Center, New York, NY; Virginia Commonwealth University, Massey Cancer Center, Richmond; American Society of Clinical Oncology, Alexandria; Virginia Cancer Center, Richmond, VA; Vanderbilt-Ingram Cancer Center, Nashville, TN; Northwest Oncology & Hematology Associates, Coral Springs, FL; Sidney Kimmel Comprehensive Cancer Center, Johns Hopkins University, Baltimore; National Cancer Institute, Bethesda, MD; Helen F. Graham Cancer Center, Newark, DE; Hematology/Oncology of Indiana, PC, Indianapolis, IN; Nebraska Cancer Specialists, PC, Omaha, NE; Samuel Oschin Comprehensive Cancer Institute, Los Angeles, CA; University of Texas, Southwestern Medical Center, Dallas, TX; British Columbia Cancer Agency, Vancouver, British Columbia, Canada; 2 天津医科大学总医院；天津市肺癌研究所；天津市肺癌转移与肿瘤微环境重点实验室

## Abstract

本文旨在为Ⅳ期非小细胞肺癌患者的治疗提供更新版推荐。本文资料检索源自2002年以来公布的相关随机试验文献。此指南范围限于化疗与生物治疗。更新委员会对这些文献进行了总结并提供了推荐更新。162篇文献符合标准被纳入参考。本推荐基于可改善总生存期的治疗方法。仅改善无进展生存期的治疗方法推动了对毒性及生存质量的监测。对于体力状态评分为0分或1分患者的一线治疗，可推荐以铂类为基础的细胞毒性药物的两药联用。对铂类治疗有禁忌的患者，可采用非铂类细胞毒性两药联合。对于体力状态评分为2分的患者，单一细胞毒性药物即可。对于疾病进展或经过4个周期的治疗仍对治疗无反应的患者，应停止一线细胞毒性化疗。即使在6个周期后患者对治疗仍有反应，亦应停止两药细胞毒性化疗。对于伴有明确的表皮生长因子受体（epidermal growth factor receptor, *EGFR*）突变的患者，可推荐一线采用吉非替尼治疗；对于EGFR突变为阴性或不明确的患者，细胞毒性化疗更佳。除具有特定临床特征的患者外，可推荐贝伐单抗与卡铂-紫杉醇联用。对于通过免疫组化证实*EGFR*阳性的肿瘤患者，可推荐西妥昔单抗与顺铂-长春瑞滨联用。多西紫杉醇、厄洛替尼、吉非替尼或培美曲塞被推荐作为二线治疗。对于未曾接受过厄洛替尼或吉非替尼治疗的患者，可推荐厄洛替尼作为三线治疗。现有数据不足以推荐常规三线采用细胞毒性药物。已有的证据也不足以推荐常规应用分子标记物选择化疗。

## 特别声明

2009年7月2日，美国食品药品监督管理局批准了培美曲塞的新指征：用于晚期非小细胞肺癌（non-small cell lung cancer, NSCLC）患者的维持治疗，当时该指南已交付印刷。最近出现了支持这一变化的数据，且未被列入此指南所综述的全部数据中。鉴于最近公布的相关数据，指南中有关维持治疗的相关推荐将会更新。

## 前言

美国临床肿瘤学会（American Society of Clinical Oncology, ASCO）于1997年首次出版了有关不可切除NSCLC的临床实践指南，并于2 0 0 3年对其进行了更新。A SCO指南不断地被审核，一旦可能会改变指南推荐的经同行评议的新研究被报道，指南即会更新。自2003年以来，大量与指南相关的文献被报道，于是ASCO决定更新指南。鉴于文献的数量所限，指南的范围仅限于Ⅳ期NSCLC的化疗和生物治疗以及有关分子标记物的评述。推荐的制定基于2002年1月-2009年5月发表的文献，且主要基于前瞻性随机对照试验（randomized controlled trial, RCT）中在总生存期（overall sur vival, OS）方面具有显著统计学差异的改善。仅改善无进展生存期（progression-free survival, PFS）的治疗方法推动了对毒性及生存质量等问题的监测。表 1为更新推荐总结。

**1:** 推荐总结

推荐序号	推荐
A.一线化疗	
推荐A1	对于ECOG/Zubrod评分体力状态为0分或1分以及可能为2分的Ⅳ期†NSCLC患者，证据支持采用化疗^*^。
推荐A2	对于体力状态评分为0分或1分的患者，证据支持两种细胞毒性药物联用作为一线治疗。由于铂类药物联用在有效率方面占优，且在OS方面具有微弱优势，故铂类药物联用优于非铂类药物联用。对铂类药物治疗有禁忌的患者，非铂类药物联用更为合理。推荐A8及A9强调了是否可将贝伐单抗或西妥昔单抗与细胞毒性药物联用作为一线治疗。
推荐A3	对于体力状态评分为2分的患者，现有数据支持采用单药化疗。对于体力状态评分为2分的患者，数据不足以推荐支持或反对两种细胞毒性药物的联用。
推荐A4	证据不支持仅基于年龄选择特定一线化疗药物或联合。
推荐A5	顺铂或卡铂均可选用。可与铂类联用的药物包括三代细胞毒性药物多西紫杉醇、吉西他滨、依立替康、紫杉醇、培美曲塞以及长春瑞滨。有证据表明，当与三代药物联用时，与卡铂相比，与顺铂联用的有效率更高且可改善生存期。与顺铂相比，卡铂似乎不易引起恶心、肾损害以及神经毒性，但容易引起血小板减少。
推荐A6	对于Ⅳ期NSCLC患者，当出现疾病进展或者在经过4个周期治疗后仍对治疗无反应时，即应停止一线细胞毒性化疗。两种细胞毒性药物联合的用药时间不宜超过6个周期。对于疾病稳定或者对一线治疗有反应的患者，证据并不支持继续使用细胞毒性化疗至疾病进展或在疾病进展之前更换化疗。
推荐A7	对于非指定患者，不应将厄洛替尼或吉非替尼与细胞毒性化疗联合作为一线治疗。在非指定患者中，证据不足以推荐单药厄洛替尼或吉非替尼作为一线治疗。对于伴有活化的*EGFR*突变的患者，可推荐采用吉非替尼作为一线治疗。如果*EGFR*突变状态为阴性或不明确，细胞毒性化疗更佳（见推荐A2）。
推荐A8	基于1项大型Ⅲ期随机对照试验的结果，指南更新委员会推荐贝伐单抗（15 mg/kg，每三周）与卡铂/紫杉醇联用，但以下患者除外：鳞癌组织学类型、伴有脑转移、临床显著的咯血、器官功能不全、ECOG体力状态评分 > 1分、接受治疗性抗凝以及临床显著的心血管疾病或药物无法控制的高血压患者。
推荐A9	基于1项大型Ⅲ期随机对照试验的结果，对于免疫组化证实EGFR阳性的肿瘤患者的一线治疗，临床医生可考虑顺铂/长春瑞滨与西妥昔单抗联用。如果可耐受，西妥昔单抗应持续使用直至疾病进展。
B.二线化疗	
推荐B1	对于体力状态良好的晚期NSCLC患者，在一线、含铂治疗过程中或之后出现疾病进展时，可选择多西紫杉醇、厄洛替尼、吉非替尼或培美曲塞作为二线治疗。
推荐B2	证据并不支持仅基于年龄选择特定二线化疗药物或联合。
C.三线化疗	
推荐C1	对于体力状态评分为0分-3分且未曾接受过厄洛替尼或吉非替尼治疗的患者，在二线治疗过程中或之后出现疾病进展时，可推荐采用厄洛替尼治疗作为三线治疗。
推荐C2	已有证据不足以推荐支持或反对采用细胞毒性药物作为三线治疗。此类患者应考虑试验性治疗、临床试验以及最佳支持疗法。
D.分子分析	
推荐D1	对于转移性NSCLC的患者，证据不足以推荐常规应用分子标记物选择系统性治疗。
推荐D2	为了获得组织以达到更为精确的组织学分类或研究目的，指南更新委员会支持通过合理的努力获得比常规细胞学标本更多的组织。
简写：NSCLC：非小细胞肺癌；ECOG：东部肿瘤协作组。 ^*^这里，术语“化疗”指任何抗癌药物，不论其作用机制（如，细胞毒性药物以及生物制剂均包括在内），除非特殊说明。 †根据国际肺癌研究会肺癌分期项目的第7版恶性肿瘤的TNM分类^[1]^。 注：本表获得© 2009 by American Society of Clinical Oncology复制许可。	

## 指南问题

### 一线化疗

**1.** 哪些Ⅳ期（根据国际肺癌研究会肺癌分期项目^[1]^的定义）NSCLC患者需要进行化疗？（注意：这里，术语“化疗”指任何抗癌药物，不论其作用机制[如，细胞毒性药物以及生物制剂均包括在内]，除非特殊说明）。

**2.** 对于Ⅳ期NSCLC患者的治疗，最有效的一线化疗是什么？在Ⅳ期NSCLC的化疗治疗中，在OS、PFS、毒性以及生存质量/症状减轻方面，获益有哪些？

**3.** 对于体力状态（performance status, PS）评分为2分的Ⅳ期NSCLC患者的治疗，最佳化疗是什么？

**4.** 对于Ⅳ期NSCLC老年患者的治疗，最佳化疗是什么？

**5.** 在Ⅳ期NSCLC的一线治疗中，顺铂是否比卡铂更有效？

**6.** 对于Ⅳ期NSCLC，一线化疗的最佳持续时间是多少？

**7.** 在Ⅳ期NSCLC的靶向治疗中，在OS、PFS、毒性以及生存质量/症状减轻等方面，获益有哪些？

### 二线化疗

**1.** 对于Ⅳ期NSCLC，是否存在最佳的二线治疗？是否有证据支持生物治疗联用作为二线治疗？在Ⅳ期NSCLC的二线治疗中，是否存在最佳的用药方案？

**2.** 对于Ⅳ期NSCLC老年患者，最佳的二线治疗是什么？

### 三线治疗

**1.** 在Ⅳ期NSCLC的治疗中，三线治疗是否起作用？

### 分子分析

**1.** 为了制定化疗方案，与之相关的组织分子分析有哪些？

## 更新方法学

### 文献综述

为了2009版更新，在MEDLINE、EMBASE以及Cochrane数据库中检索2002年-2008年7月符合指南更新规程纳入标准的研究。简言之，凡符合以下条件的治疗研究均可纳入：治疗研究将Ⅳ期NSCLC患者随机分为一种化疗组和另一种化疗组、化疗组和最佳支持疗法（best supportive care, BSC）组或化疗组和安慰剂组，并且报道了疗效结果（OS、PFS或客观有效）、毒性或健康相关的生存质量（health-related quality of life, HRQOL）/症状缓解。对于细胞毒性化疗，检索仅限于Ⅲ期RCT；对于生物制剂，检索包括Ⅱ期和Ⅲ期随机试验；对于分子分析，检索包括Ⅱ期和Ⅲ期试验、队列研究以及临床试验的回顾性亚组分析。该系统评价由加拿大癌症疾病组循证中心开始实施，并由ASCO完成。为了对文献综述更新方法学及过程进行充分了解，可登录www.asco.org/guidelines/nsclc，参考该指南的网络版。

### 专业委员会的组成及基于循证的共识形成

ASCO临床实践指南委员会组成了一个更新委员会，其中包括学术以及社区肿瘤内科专家、统计学专家以及两个患者代表。委员会的所有成员均参与了指南草案的筹备工作，指南随后被全体委员会审阅。该指南随后又被提交至*Journal of Clinical Oncology*进行同行评议。指南及稿件的内容在发表前已经临床实践指南委员会及ASCO董事会评议且获批准。

## 结果

### 文献综述总结

自上一版更新以来，系统评价共检索出190篇文献，这些文献或与第1版中的至少一个临床问题相关，或发表了更新结果。并非所有的指南问题都得到了新的数据。在190篇文献中，94篇报道了化疗，23篇报道了生物治疗，73篇报道了分子分析。另外，在系统评价完成之后从出版物、摘要以及报告中提取数据。推荐基于52项RCT和29项*meta*分析（MA），及所纳入的仅用于分子分析的回顾性组织学分析。达到合格标准且被整个指南更新纳入的研究见表 2。

**2:** 指南纳入且满足合格标准的研究

问题	干预	新研究
Ⅳ期NSCLC患者的一线化疗		
A1.哪些Ⅳ期NSCLC患者需要进行化疗？	化疗*v* BSC	1项RCT^[3]^；1项MA^[2]^。
A2.对于Ⅳ期NSCLC患者的治疗，最有效的一线化疗是什么？	铂类*v*非铂类化疗；两种细胞毒性药物*v*一种细胞毒性药物*v*三种细胞毒性药物；紫杉醇/卡铂每周方案*v*每三周方案。	17项RCT^[4-7, 9-19, 25, 134]^； 5项MA^[8, 20-23]^； 2项预先计划的亚组分析^[26, 27]^。
A3.对于PS为2分的患者的治疗，最佳化疗是什么？	一种或两种细胞毒性药物*v*一种或两种药物（包括EGFR TKIs）。	2项RCT^[29, 30]^； 2项RCT的亚群分析^[5, 28]^。
A4.对于老年患者的治疗，最佳化疗是什么？	一种或两种细胞毒性药物*v*一种或两种细胞毒性药物；对于年龄低于及长于分割点年龄的患者，治疗相同。	3项RCT^[28, 32, 33]^；2项亚组分析^[5, 34]^； 1项回顾性分析^[35]^；1项汇总分析^[3]^。
A5.顺铂是否比卡铂更有效？	顺铂*v*卡铂	9项RCT^[24, 40-47]^；3项MA^[37-39]^。
A6.对于Ⅳ期NSCLC，一线治疗的最佳持续时间是多少？	不同的治疗周期的治疗；维持化疗；即刻*v*延迟交替化疗。	6项RCT^[48-53]^。
A7.在靶向治疗过程中，在总生存期、无进展生存期、毒性以及生存质量/症状缓解方面，获益有哪些？		
推荐A7	化疗*v*化疗联合EGFR TKI；化疗*v* EGFR TKI。	6项RCT^[30, 54-58]^。
推荐A8	贝伐单抗联合双药化疗*v*双药化疗。	3项RCT^[31, 59-61]^；1项RCT亚组分析^[63]^。
推荐A9	西妥昔单抗联合双药化疗*v*双药化疗。	4项RCT^[64-67]^。
Ⅳ期NSCLC患者的二线治疗		
B1.对于Ⅳ期NSCLC，是否存在最佳二线治疗？是否有证据支持生物治疗联用作为二线治疗？在二线治疗中，是否存在最佳用药方案？	培美曲塞*v*多西紫杉醇；EGFR TKI *v*安慰剂/BSC；吉非替尼*v*多西紫杉醇；贝伐单抗/厄洛替尼*v*厄洛替尼或*v*化疗或*v*贝伐单抗/化疗；多西紫杉醇每周方案*v*多西紫杉醇每三周方案。	11项RCT^[62, 71-76, 80-83]^； 2项亚组分析^[77, 78]^； 1项SR^[70]^； 1项IPDMA^[69]^。
B2.对于老年患者，最佳的二线治疗是什么？	多西紫杉醇*v*培美曲塞，基于年龄。	1项RCT的回顾性亚组^[135]^。
Ⅳ期NSCLC患者的三线化疗		
C.三线治疗是否起作用？		
推荐C1	厄洛替尼*v*安慰剂。	1项RCT^[74]^。
推荐C2	三线或四线化疗。	1项回顾性分析^[84]^。
分子分析		
D1.为了制定化疗方案，与之相关的Ⅳ期NSCLC组织分子分析有哪些？		
*EGFR*	吉非替尼、厄洛替尼、化疗。	37项分析(包括5项临床试验) ^[68, 85-120]^。
		1项SR^[79]^；1项IPDMA^[69]^。
*KRS*	吉非替尼、厄洛替尼、化疗。	7项分析^[87, 88, 91, 92, 100, 121]^。
*ERCC1*/*RRM1*	化疗：顺铂、吉西他滨、其它。	7项分析^[122-128]^。
*VEGF*	化疗、贝伐单抗。	5项分析^[129-133]^。
简写：NSCLC：非小细胞肺癌；RCT：随机对照试验；MA：*meta*分析；PS：体能状态；EGFR：表皮生长因子受体；TKI：酪氨酸激酶抑制剂；BSC：最佳支持疗法；SR：系统评价；IPDMA：独立患者数据*meta*分析。 注：本表获得© 2009 by American Society of Clinical Oncology复制许可。		

### 文献制约

纳入PS较差（根据东部肿瘤协作组[ECOG]/Zubrod评分PS≥2分或根据Karnofsky PS评分 < 70%）患者或老年患者的试验数量有限。另外，目前缺乏采用三线及以上治疗的患者的Ⅲ期数据。指南中的推荐基于前瞻性RCT结果中具有显著统计学意义的改善，主要为OS。因此，在评审过程中，治疗选择中的一些重要问题，包括毒性、生存质量以及成本效益，被更新委员会忽视。关于一些临床问题，仅观察到PFS的改善。仅改善PFS的治疗方法推动了对毒性、不良事件（adverse event, AE）以及生存质量的监测。

## Ⅳ期NSCLC指南更新推荐

一线化疗推荐从A开始，二线化疗从B开始，三线化疗从C开始，分子分析从D开始。

### 一线化疗

**1.** 临床问题。哪些Ⅳ期NSCLC患者需要进行化疗？

2003版推荐。化疗适用于指定的Ⅳ期NSCLC患者。化疗可延长Ⅳ期NSCLC患者生存期，且最适合PS良好（ECOG/Zubrod评分PS为0分或1分以及可能为2分）的患者。如果Ⅳ期NSCLC患者需进行化疗，则只有患者PS良好时才可开始。

2009版推荐A1。对于ECOG/Zubrod评分PS为0分、1分以及可能为2分的Ⅳ期NSCLC患者，证据支持采用化疗。

文献更新。该推荐的结论与2003版指南更新一致。自上一版指南更新公布以来，1项仅在NSCLC患者中比较化疗与支持疗法的试验的MA已更新^[2]^。该MA比较了化疗与BSC的疗效，结果显示化疗可降低死亡风险（危险比=0.77；95%CI: 0.71-0.83；*P*≤0.000 1）并可增加1年生存率。该MA包括16项试验，共2 714例患者；12项试验采用了含铂方案，13项试验报道了PS。该MA发现，PS为2分的患者亦可获益，尽管获益低于PS为0分-1分的患者。

自上一版更新以来^[3]^，在1项比较化疗联合BSC与单一BSC的涉及725例患者的RCT中，最常见的3级或4级AE为血液系统AE、恶心以及呕吐。罕见但严重的AE包括神经毒性和肾毒性。

**2.** 临床问题。对于Ⅳ期NSCLC患者的治疗，最有效的一线化疗是什么？

2003版推荐。晚期NSCLC患者的一线化疗应采用两药联合方案。在一线治疗中，不含铂化疗方案可作为含铂方案的候补。

2009版推荐A2。对于PS为0分或1分的患者，证据支持两种细胞毒性药物联用作为一线治疗。由于铂类药物联用在有效率方面占优，且在OS方面具有微弱优势，故铂类药物联用优于非铂类药物联用。对铂类药物治疗有禁忌的患者，非铂类药物联用更为合理。推荐A8及A9强调了是否可将贝伐单抗或西妥昔单抗与细胞毒性药物联用作为一线治疗。

文献更新。自上一版指南公布以来，有4项试验^[4-7]^和1项MA^[8]^比较了两药联用与单一药物的疗效，结果显示，接受两药联用治疗的患者在影像学反应率方面有改善，且其中1项试验^[7]^以及MA^[8]^发现OS方面的改善具有显著的统计学意义。单个临床试验纳入的患者数为289^[4]^-561^[5]^。检测三种细胞毒性药物联用与两种药物联用的个体试验或MA均未发现三种细胞毒性药物联用可改善生存期，但均显示三种细胞毒性药物联用可使毒性AE增加^[8-11]^。这些数据验证了上一版推荐关于两种细胞毒性药物联用的获益，且亦显示两种细胞毒性药物联用优于三种细胞毒性药物联用。

自2003版指南以来，比较含铂方案与不含铂方案的15项RCT^[4-7, 9-19]^以及4篇基于文献的MA^[20-23]^已发表。MA纳入的患者数为2 351^[20]^-7 633^[21]^，单个RCT纳入的患者数为281^[17]^-1 725^[21]^。7项试验^[4-7, 9, 10, 15]^及4项MA^[20-23]^表明，与不含铂方案相比，含铂治疗在有效率方面具有显著的统计学优势，且4项MA^[20-23]^及1项单个研究^[7]^表明采用含铂治疗可带来显著的生存优势。

据报道，铂类药物的毒性更高。铂类药物特异的AE包括肾毒性及胃肠道问题。12项单个试验^[4-7, 10-14, 16, 17, 36]^表明，铂类治疗组的血液系统毒性显著增高，且7项试验^[4, 5, 9-11, 13, 15]^表明铂类治疗组的非血液系统毒性显著增高。含铂治疗的禁忌症包括：对顺铂或卡铂过敏、基线听力丧失、肾功能不全、尽管使用了最佳预防呕吐措施仍伴有不可耐受的恶心（参见A SCO指南中止吐药^[156]^）、不能耐受用于预防呕吐的糖皮质激素以及患者拒绝服用铂类药物。对于这些患者，可选择非铂类联合。

指南的该部分内容综述了任一用药方案的相关数据。证据仅限于有关紫杉醇/卡铂方案的2项研究^[24, 25]^，且表明紫杉醇/卡铂每周方案与每三周方案间无差异。每三周方案的血液系统毒性更为严重。

与基于其它药物的联用相比，一些基于顺铂的联用效果较好。基于单个临床试验或回顾性（尽管已预先计划）亚组分析^[26, 27]^发现，对于总体NSCLC人群，多西紫杉醇/顺铂优于长春瑞滨/顺铂；对于非鳞癌NSCLC患者，培美曲塞/顺铂优于吉西他滨/顺铂；对于鳞癌NSCLC患者，吉西他滨/顺铂优于培美曲塞/顺铂。现有数据不足以将含铂两药联用的选择局限于仅基于疗效的两种选择，且临床医生须基于其它因素，包括用药方案和AE，选择优于其它化疗方案的方案。

**3.** 临床问题。对于PS为2分的Ⅳ期NSCLC患者的治疗，最佳化疗是什么？

2003版推荐。对于老年患者或ECOG/Zubrod评分PS为2分的患者，现有证据支持使用单药化疗。

2009版推荐A3。对于PS为2分的患者，现有数据支持采用单药化疗。对于PS为2分的患者，数据不足以推荐支持或反对两种细胞毒性药物的联用。

文献更新。自2003版指南更新以来，该推荐未做更改。自上一版更新，探讨单药和两药联用治疗PS为2分患者的新证据包括2项基于PS的有计划亚组分析的Ⅲ期试验^[5, 28]^、1项仅针对PS为2分患者的Ⅲ期试验^[29]^、1项在PS为2分的患者中比较传统细胞毒性化疗与厄洛替尼的Ⅱ期试验^[30]^。此推荐亦部分基于至少1项排除PS为2分的患者的新研究证据^[31]^。4项分析报道了生存结果^[5, 28-30]^，其中3项报道了PFS或进展时间（TTP）^[5, 28, 29]^；所有4项分析均报道了有效率。纳入的患者数为103^[30]^-561^[5]^。

1项RCT和1项亚组分析发现，对于PS为2分的患者，比较组间无生存获益（两药联用*v*两药联用^[29]^和单一用药*v*两药联用^[28]^）；而其它亚组分析和其它RCT发现，与单一用药相比，两药联用可使PS为2分的患者生存获益^[5, 30]^。基于PS分层且报道AE的唯一分析发现，单一用药与两药联用间无差异^[5]^。

鉴于归类为PS为2分的患者间的异质性、PS评分的主观性以及支持最佳化疗方案的一致性数据的缺乏，对PS为2分的患者，更新委员会不推荐两种细胞毒性药物联用，而且更新委员会发现，有些归类为PS为2分的患者可能无法耐受单药化疗。

**4.** 临床问题。对于Ⅳ期NSCLC老年患者的治疗，最佳化疗是什么？

2003版推荐。对于老年患者或ECOG/Zubrod评分PS为2分的患者，已有数据支持使用单药化疗。

2009版推荐A4。证据不支持仅基于年龄选择特定的一线化疗药物或联用。

文献更新。自上一版指南更新公布以来，更新的证据包括3项新的RCT^[28, 32, 33]^、2项新的亚组分析^[5, 54]^、1项回顾性分析^[35]^以及1项强调该问题的汇总分析^[36]^。这些研究探讨了65岁或70岁以上老年患者（各研究对老年人的定义不同）的最佳一线治疗。这些试验及3项分析（非MA）中纳入的老年患者数为67^[35]^-401^[34]^。

3项分析^[5, 34, 35]^及1项汇总分析^[36]^的结果均未发现定义为老年与定义为年轻的患者在生存期方面存在差异。另外，2项试验在65岁或70岁以上的老年患者中比较了两种方案^[28, 32]^。所有证据表明，对于老年患者来说，两种方案在生存期方面无差别。其中4项研究报道了毒性，有2项研究发现，相对于单一用药，两药联用的毒性更为严重^[28, 32]^。

总之，自2003版更新以来的临床试验数据充实了这一推荐：对于Ⅳ期NSCLC患者，不宜仅依据年龄选择化疗。相对于年轻患者，细胞毒性化疗对老年患者的毒性更大，但可能也会得到同等的获益。

**5.** 临床问题。在Ⅳ期NSCLC的一线治疗中，顺铂是否比卡铂更有效？

2003版推荐。2003版推荐中并未特别强调该问题。

2009版推荐A5。顺铂或卡铂均可选用。可与铂类联用的药物包括第三代细胞毒性药物多西紫杉醇、吉西他滨、依立替康、紫杉醇、培美曲塞以及长春瑞滨。有证据表明，当与第三代药物联用时，与卡铂相比，与顺铂联用的有效率更高且可改善生存期。与顺铂相比，卡铂似乎不易引起恶心、肾损害以及神经毒性，但容易引起血小板减少。

文献更新。推荐基于文献中所有药物在OS、毒性或者生存质量方面均缺乏一致性优势。文献检索共检索出3项MA^[37-39]^以及9项比较顺铂、卡铂与各种其它细胞毒性药物联用的单个RCT^[24, 40-47]^。单个RCT纳入的患者数为153^[43]^-1 218^[26]^。2项基于文献的MA^[38, 39]^以及1项单个患者数据MA分析（IPDMA）^[37]^发现，顺铂的有效率显著优于卡铂。3项MA和3项单个试验均未发现顺铂与卡铂在生存期方面存在显著差异^[42, 46, 47]^。在MA中，当与第三代药物联用时，在包括非鳞癌NSCLC的特定亚组中，顺铂在生存期方面优于卡铂。

与顺铂相比，卡铂更容易增加骨髓抑制作用，并引起血小板减少，但不易引起恶心。数项单个试验表明卡铂较顺铂更易引起血小板减少^[20, 37, 39, 47]^。紫杉烷类为神经毒性最强的药物之一，卡铂与紫杉烷类联用试验的优势削弱了有关神经毒性的数据^[46]^。与卡铂相比，顺铂更易引起耳毒性及外周神经毒性。这些毒性的风险可将顺铂、卡铂或两者排除。例如，顺铂的相对禁忌症包括基线听力丧失、肾功能不全、合并性疾病（如限制静脉生理盐水输入的充血性心力衰竭、泌尿系统问题；可限制用于预防呕吐的糖皮质激素使用的糖尿病）。卡铂的相对禁忌症包括基线血小板减少和出血危险。

总之，顺铂在疗效结果方面优于卡铂；卡铂毒性更小。当为患者推荐顺铂或卡铂时，医生们应该考虑患者的个体因素。

**6.** 临床问题。对于Ⅳ期NSCLC患者，一线治疗的最佳持续时间是多少？

2003版推荐。对于Ⅳ期NSCLC患者，如4个周期时患者对治疗仍无反应，应停止一线化疗。委员会一致认为，对于Ⅳ期NSCLC患者，一线化疗的用药时间不应超过6个周期。

2009版推荐A6。对于Ⅳ期NSCLC患者，当患者疾病进展或4个周期后患者对治疗仍无反应，应停止一线细胞毒性化疗。两种细胞毒性药物联用的用药时间不应超过6个周期。对于疾病稳定或对一线治疗有反应的患者，证据并不支持继续细胞毒性化疗至疾病进展或者疾病进展前更换化疗方案。

文献更新。推荐基于自上一版更新以来发表的4项RCT^[48-51]^。另外，有2项关于继续用药或更换化疗方案的RCT（仅以摘要形式发表）^[52, 53]^。在4项已发表的有关持续时间的RCT中，纳入的患者数为230^[50]^-452^[49]^。这些研究显示，追加/延长持续时间 > 4个周期未见显著生存优势。其中2项研究发现，追加化疗后PFS或TP显著延长。在报道HRQOL的3项试验中^[49-51]^，其中1项试验发现，在HRQOL方面，在某一标准上单一用药方案6个周期的治疗优于3个周期^[49]^；1项试验发现，在另一标准上单一用药方案4个周期的治疗优于6个周期^[51]^。在延长化疗时间后，外周神经病变^[50]^及血小板减少症更为严重^[51]^；然而，研究并未报道短疗程与长疗程在毒性方面存在其它显著性差异。

研究表明，对于NSCLC患者的治疗，非交叉耐药药物的即刻序贯或交替使用并不优于最佳一线联合化疗^[137]^。随着一线化疗后可改善患者生存期的二线药物的问世，人们对一线治疗结束后立即使用非交叉耐药药物治疗是否可改善生存期重新燃起兴趣。2项RCT均未发现该策略可使生存期显著改善；在该2项研究中，瞬时服用非交叉耐受药物使PFS显著改善；然而，整个指南指出这些研究可能存在偏差^[52, 53]^。更新委员会预测了有关厄洛替尼作为维持治疗的2项研究的结果。

总之，在体现生存优势的更为充分的数据出现之前，这些数据表明，在4个周期后持续有效的化疗或即刻启用交替化疗可改善PFS，而不是OS。然而，追加细胞毒性化疗导致的AE的增加可削弱PFS的改善。

**7.** 临床问题。在Ⅳ期NSCLC患者的靶向治疗中，在OS、PFS、毒性以及生存质量/症状减轻等方面，获益有哪些？

2003版推荐。在研药物或方案的初始治疗适宜指定的Ⅳ期NSCLC患者，并且如果2个周期治疗后患者仍无反应，即可更换为另一种有效的治疗方案。

2009版推荐A7。对于非指定的Ⅳ期NSCLC患者，厄洛替尼或吉非替尼不应与细胞毒性化疗联用作为一线治疗。对于非指定的患者，现有数据不足以推荐单药厄洛替尼或吉非替尼作为一线治疗。对于伴有活化*EGFR*突变的患者，可推荐一线使用吉非替尼。如果*EGFR*突变状态为阴性或不明确，则细胞毒性化疗较好（见推荐A2）。

文献更新。自上一版更新，共有6项该主题相关的RCT^[30, 54-58]^。4项试验研究了厄洛替尼或吉非替尼与细胞毒性化疗两药联用作为一线治疗的疗效^[54-57]^。纳入的患者数为1 037^[56]^-1 172^[57]^。所有的4项试验均报道了疗效结果且发现将EGFR赖氨酸激酶抑制剂（TKI）加入化疗并未改善生存期、PFS或有效率。4项研究中的3项研究均显示，与EGFR TKI联用组的AE更多（主要为皮肤毒性和腹泻）。

有关厄洛替尼作为一线单一疗法的唯一文献为1项小型Ⅱ期试验，该试验在PS为2分的患者中比较了厄洛替尼与卡铂/紫杉醇的疗效^[30]^。与厄洛替尼单药相比，两药联合化疗具有显著的生存优势，尽管PFS和总有效率并不存在显著差异。并未根据*EGFR*突变状态对患者进行筛选。

在Ⅲ期艾瑞莎泛亚洲研究（IPASS）中，调查者在东亚人群中比较了吉非替尼与卡铂/紫杉醇作为一线治疗的疗效，所有患者均为腺癌以及轻微或从不吸烟者^[58]^。主要终点为PFS，吉非替尼组PFS显著延长；OS为次要终点，未见显著延长。化疗组的血液系统AE、脱发、神经病变及恶心比较严重，而吉非替尼组的腹泻及皮肤毒性比较严重。通过*EGFR*突变状态对PFS进行分析，结果显示，伴有突变的患者采用吉非替尼治疗获益较大，而未伴有突变的患者采用化疗获益较大。大多数患者肿瘤的*EGFR*突变状态为阴性或不明确，这些患者可使用细胞毒性化疗（见推荐A2）。

2009版推荐A8。基于1项大型的Ⅲ期RCT的结果，更新委员会推荐贝伐单抗（15 mg/kg，每三周一次）与卡铂/紫杉醇联用，但以下患者除外：鳞癌组织学类型、脑转移、临床显著的咯血、器官功能不全、ECOG PS评分 > 1分、接受治疗性抗凝、临床显著的心血管疾病或药物无法控制的高血压患者（根据Sandler等^[31]^实施的登记试验的排除标准）。如果患者可耐受，贝伐单抗可持续使用直至疾病进展。

文献更新。3项有关抗血管形成药贝伐单抗的RCT（2项Ⅲ期）已发表^[31, 59-61]^。纳入的患者数为99^[59]^-1 043^[61]^。其中，2项研究报道了OS和PFS^[31, 59]^，1项仅报道了PFS^[61]^。贝伐单抗与卡铂/紫杉醇联用且报道了OS的Ⅲ期试验发现，OS、PFS以及有效率均显著改善^[31]^。贝伐单抗与顺铂/吉西他滨联用的第2项Ⅲ期试验验证了PFS的获益，但在调查者公布试验结果时仍未得到生存结果的分析^[61]^。

贝伐单抗的主要AE包括4级和5级血液系统毒性，以及非血液系统毒性。在特定肿瘤的Ⅱ期试验中，首先可见严重的出血，这使得Ⅲ期试验的纳入标准仅限于非鳞癌患者，且符合推荐A8中所列的任一标准的患者亦被排除在外。在所有试验中毒性死亡事件均有发生，包括1项贝伐单抗联合化疗或厄洛替尼以及不联合化疗或厄洛替尼作为二线治疗的试验^[62]^。由于贝伐单抗与吉西他滨、顺铂联用并未改善OS，及贝伐单抗与其它细胞毒性方案联合的Ⅲ期数据的缺乏，现有数据不足以使更新委员会推荐贝伐单抗与细胞毒性化疗方案联用（而不是卡铂/紫杉醇）。尽管PFS和总有效率得到改善，但贝伐单抗的毒性增加削弱了这种改善。基于1项主要Ⅲ期试验的亚组分析，在老年人群中应特别关注贝伐单抗的毒性，该分析显示，老年亚组的毒性增加且OS并无明显改善^[63]^。化疗与贝伐单抗联用的最佳持续时间尚未明确。

2009版推荐A9。基于1项大型Ⅲ期RCT的结果，对于经免疫组化（IHC）证实EGFR阳性的肿瘤患者的一线治疗，临床医生可考虑西妥昔单抗与顺铂/长春瑞滨联用。如果患者可耐受，西妥昔单抗应持续使用直至疾病进展。

文献更新。4项RCT比较了西妥昔单抗与化疗联合与单独化疗的疗效^[64-67]^。在包括1 125例患者的Ⅲ期FLEX（艾比特思肺癌一线治疗）研究中，顺铂/长春瑞滨联合西妥昔单抗组患者的中位OS比顺铂/长春瑞滨组延长1.2个月（*P*=0.044 1），尽管两组间PFS相同^[67]^。在另一项Ⅲ期试验的摘要中，西妥昔单抗联合化疗可使PFS显著延长，但生存期未见改善^[66]^。FLEX试验的纳入标准为经IHC明确的EGFR蛋白表达阳性^[67]^。不符合系统评价的1项Ⅱ期试验的相关研究发现，伴有EGFR扩增（通过荧光原位杂交明确）患者的PFS及中位OS均改善^[68]^。这些结果尚未在前瞻性研究中得到证实。

西妥昔单抗联合化疗常见的AE包括发热性嗜中性粒细胞减少、痤疮/皮疹、腹泻以及输液相关反应。这些研究结果提示，当与顺铂/长春瑞滨联用时，西妥昔单抗可增加生存期获益。相对于贝伐单抗，证据不足以推荐西妥昔单抗与其它化疗方案联用。基于这些研究的设计，可推荐西妥昔单抗持续用药至患者不可耐受或疾病进展。然而，西妥昔单抗与化疗联合治疗的最佳持续时间尚不明确。

### 二线化疗

**1.** 临床问题。对于Ⅳ期NSCLC，是否存在最佳的二线治疗？是否有证据支持生物治疗联用作为二线治疗？在Ⅳ期NSCLC的二线治疗中，是否存在最佳用药方案？

2003版推荐。对于在一线、基于铂类药物治疗中出现疾病进展且PS良好的局限性晚期或转移性NSCLC患者，可推荐多西紫杉醇作为二线治疗。局限性晚期或转移性NSCLC患者在基于铂类药物及多西紫杉醇治疗均失败后，可推荐采用吉非替尼。

2009版推荐B1。对于一线、基于铂类药物治疗过程中或之后出现疾病进展且PS良好的晚期NSCLC患者，多西紫杉醇、厄洛替尼、吉非替尼或培美曲赛均可作为二线治疗。

文献更新。该推荐涉及细胞毒性化疗及靶向治疗。推荐基于9项新的Ⅲ期RCT、2项新的Ⅱ期RCT、1项新的IPDMA^[69]^、1项新的系统评价^[70]^以及2项有关Ⅲ期试验的亚组分析，该2项亚组分析表明多西紫杉醇、厄洛替尼、吉非替尼或培美曲赛作为二线化疗可带来整体获益。有关化疗的研究包括5项新的Ⅲ期RCT^[71-75]^、1项Ⅱ期RCT^[76]^、2项临床试验的回顾性分析^[77, 78]^、1项系统评价^[79]^以及1项IPDMA^[80]^；有关联合生物治疗的研究包括1项Ⅱ期RCT^[62]^以及1项Ⅲ期RCT^[80]^；有关服药方案的研究包括3项Ⅲ期试验^[81-83]^。7项RCT比较了新型治疗（伴有或不伴有标准治疗）与标准治疗或新型靶向治疗与安慰剂^[62, 72-76, 80]^的疗效。培美曲塞与多西紫杉醇进行了比较^[72]^，厄洛替尼与安慰剂进行了比较^[74]^，吉非替尼与BSC/安慰剂^[75]^以及与多西紫杉醇进行了比较^[73, 76]^。纳入的患者数为120-1 692^[62, 75]^。2003版更新数据与1项新的系统评价推荐使用多西紫杉醇。在与治疗相关且达主要疗效终点的8项新的二线RCT中，其中1项显示OS显著改善^[74]^，4项研究显示优势无差异^[71-73, 76]^。3项试验结果为阴性；其中2项试验采用贝伐单抗与厄洛替尼联合；另1项试验比较了吉非替尼与安慰剂的疗效^[62, 75, 80]^。

4项研究比较了多西紫杉醇不同剂量或用药方案的疗效^[71, 81-83]^。比较用药方案的3项试验表明每周方案并无生存优势（与每三周方案相比）^[81-83]^；通常每三周方案的血液系统毒性更为严重。

在比较多西紫杉醇与新型制剂疗效的研究中，多西紫杉醇的AE包括嗜中性粒细胞减少、发热性嗜中性粒细胞减少、须用粒细胞集落刺激因子、腹泻以及脱发。厄洛替尼的最常见AE为皮疹和腹泻。另外，也可见间质性肺疾病，但发生率比较低^[55]^。培美曲塞的常见AE为嗜中性粒细胞减少、发热性嗜中性粒细胞减少、须用粒细胞集落刺激因子，但较多西紫杉醇少见。吉非替尼的常见AE为皮疹和腹泻；另外，也可见间质性肺疾病，但发生率比较低。

**2.** 临床问题。对于Ⅳ期NSCLC的老年患者，最佳二线治疗是什么？

2003版推荐。2003版推荐中并未特别强调该问题。

2009版推荐B2。证据并不支持仅基于年龄选择特定的二线化疗药物或药物联用。

文献更新。自上一版指南公布以来，1项有关大于70岁的Ⅳ期NSCLC患者二线化疗的新的回顾性RCT亚组分析已发表，该分析基于年龄比较了多西紫杉醇与培美曲塞的疗效^[135]^。571例患者参与了培美曲塞的登记试验，上述亚组分析即来自该登记试验。≥70岁患者从两种制剂中的获益相近。服用多西紫杉醇的≥70岁患者与 < 70岁患者在生存期方面的差异并不显著（中位OS，分别为7.7个月*v* 8.0个月；*P*=不显著）。服用培美曲塞的≥ 70岁患者的OS（并不显著）及TP较 < 70岁患者长（中位OS，分别为9.5个月*v* 7.8个月；*P*=不显著）。毒性并不随年龄而显著改变。

### 三线治疗

**1.** 临床问题。在Ⅳ期NSCLC治疗中，三线治疗是否有作用？

2003版推荐。2003版推荐中并未特别强调该问题。

2009版推荐C1。对于未曾接受过厄洛替尼或吉非替尼治疗且PS为0分-3分的患者，在二线化疗过程中或之后出现疾病进展时，可推荐厄洛替尼作为三线治疗。

文献更新。该推荐基于1项比较厄洛替尼与安慰剂疗效的RCT，这是推荐厄洛替尼作为二线治疗选择的基础（见推荐B1）^[74]^。该试验纳入的患者均曾接受过一个或两个方案的治疗。相对于作为二线治疗，厄洛替尼作为三线治疗同样有效。在生存期的多因素分析中，曾接受过的治疗方案数无显著差异。总之，有关曾接受过两个化疗方案患者的临床试验较少。

2009版推荐C2。已有的证据不足以推荐支持或反对细胞毒性药物作为三线治疗。患者应考虑临床试验、试验性治疗或BSC。

文献更新。目前尚无支持在三线治疗中常规使用细胞毒性化疗的Ⅲ期证据。因此，该推荐参考1项回顾性分析的结果。该分析共纳入700例曾接受过两个或以上化疗方案（包括至少一个铂类药物和多西紫杉醇方案）的再发NSCLC患者^[84]^。研究发现，后续方案的生存期和有效率均降低。接受三线和四线细胞毒性治疗的患者的有效率较低，有效期较短，且毒性较大。对于这些患者，除试验性治疗和临床试验外，仅支持疗法为合理选择。

### 分子分析

**1.** 临床问题。为了制定化疗方案，与之相关的组织分子分析有哪些？

2003版推荐。对于晚期、不可切除的肿瘤患者，NSCLC组织学类型并不是重要的预后因素。新的、假定的预测因子如*RAS*突变或*p53*突变的应用尚处于研究中，未应用于制定临床决策。

2009版推荐D1。现有数据不足以推荐常规应用分子标记物选择转移性NSCLC化疗药物。

文献更新。这是指南更新中一个新的主题。指南综述了超过5个同行评议的出版物中的分子标记物。系统评价发现，≥5个出版物中的标记物如下：*EGFR*、*KRAS*、ERRC1/RRM1和及VEGR。

*EGFR*。多项研究已试图阐明EGFR状态是否可用于预测对EGFR TKIs的反应。可通过3种主要的方法评估肿瘤中EGFR的状态：通过IHC测定蛋白水平；例如通过荧光原位杂交测定DNA拷贝数目水平；以及通过突变分析测定DNA序列水平。所有检测均在石蜡包埋的肿瘤块中进行，但突变分析需要提取DNA。

文献包括研究EGFR状态及主要研究EGFR TKIs应用（大部分为吉非替尼）的37项报道^[68, 85-12]^。另外，综述包括1项系统评价^[79]^和1项IPDMA^[69]^。每一研究中分析的肿瘤数为20^[98]^-731^[104]^。24项研究为回顾性的，其中包括涉及吉非替尼和厄洛替尼的主要临床试验的5项分析。在该5项分析中^[87, 89, 91, 94, 104]^，肿瘤标本收集未被授权，且检测肿瘤生物标记物患者的比例较低。有3项*meta*分析或汇总分析^[69, 79, 92]^；5项临床试验^[88, 90, 105, 114]^，包括1项Ⅲ期RCT^[58]^；以及4项有关临床试验的回顾性分析^[68, 91, 104, 113]^。

31项研究报道了OS。31项中的20项研究通过突变状态报道了OS，31项中的14项表明伴有*EGFR*突变阳性（通过3种方法中的任一方法检测）的肿瘤患者获益显著。31项中的28项研究报道了PFS（或TTP）；31项中的21项研究通过突变状态报道了PFS，其中16项研究显示伴有突变阳性的肿瘤（通过3种方法中的任一方法检测）患者获益显著。

基于临床显著的PFS的改善、较好的毒理参数以及生存质量的改善，对于*EGFR*突变的NSCLC患者，推荐A7支持一线使用吉非替尼，而非卡铂和紫杉醇。这些数据表明试图检测NSCLC肿瘤中*EGFR*突变状态是有道理的。然而，至今仍无研究表明基于分子标记物选择化疗可改善OS。

*KRAS*。*KRAS*占肺腺癌中RAS突变的90%，且NSCLC中大约97%的KRAS突变涉及密码子12或13；在鳞癌中突变较为罕见^[138, 139]^。多项研究试图阐明*KRAS*突变是否可作为对EGFR TKIs缺乏反应的预测性生物标记物。文献检索共检索出涉及*KRAS*试验的7篇文献^[87, 88, 91, 92, 100, 121, 157]^。分析的肿瘤数为41^[88]^-274^[87]^。2项研究为RCT的回顾性分析^[87, 91]^，2项为回顾性组织分析^[100]^，1项为扩展项目中患者的前瞻性分析^[121]^，1项为汇总分析^[92]^，且1项为≥70岁患者的前瞻性Ⅱ期单组试验（single-arm）的相关研究^[88]^。所有研究均报道了*KRAS*的突变率。这些分析研究了不同化疗药物及EGFR TKIs的有效率。7项分析中的6项报道了有效率，3项报道了中位生存期，4项报道了PFS/TTP。对于伴有*KRAS*突变的肿瘤患者，无一分析报道了厄洛替尼或吉非替尼在有效率、生存期或PFS方面的获益。伴有*KRAS*突变的肿瘤患者对EGFR TKIs的有效率接近零。目前尚缺乏前瞻性Ⅲ期数据。

*ERCC1*/*RRM1*。影响化疗药物代谢与疗效的分子包括：核糖核酸酶还原酶1（ribonucleotide reductase subunit 1, RRM1），其对核酸代谢非常重要而且是吉非替尼疗效的优势分子决定簇；切除修复交叉互补基因1[excision repair cross-completion (or cross-complementation) group 1, ERRC1]，为对铂类诱导的DNA加合修复非常重要的核酸切除修复复合体的组分。指南综述了有关这些标记物的7篇文献^[122-128]^，包括4项有关临床试验的回顾性分析，2项源自RCT的前瞻性分析，以及1项相关分析。纳入的患者数为53^[127]^-324^[123]^。研究中的化疗药物包括顺铂、卡铂、紫杉醇、多西紫杉醇、吉西他滨、长春瑞滨以及异环磷酰胺。研究表明，低水平的标记物可能对化疗获益具有预测作用；然而，目前尚无充足的前瞻性Ⅲ期数据以推荐这些标记物的应用。

*VEGF*。血管内皮生长因子（vascular endothelial growth factor, VEGF）是肿瘤血管形成与通透性中重要的血管形成因子。在多种癌症中可观察到VEGF的过表达且通常其过表达与无复发生存期和OS较差有关。贝伐单抗为一种直接拮抗VEGF的单克隆抗体，研究表明其可给NSCLC患者带来临床获益。VEGF的水平通常可通过酶联免疫吸附试验测定血清或血浆中的含量而确定。文献检索共检索出在血清中分析VEGF的5项研究；4项前瞻性研究（并非均为RCT），且在该4项试验中所有患者均接受化疗治疗（如，患者均未接受贝伐单抗治疗）^[129-133]^。所有的4项研究均报道了有效率，然而仅2项研究报道了OS。纳入的患者数为21^[130]^-160^[129]^。1项存在显著性差异的研究发现，低水平VEGF患者的生存时间几乎为高水平（> 500 pg/mL）患者的两倍^[130]^。第5项VEGF研究为ECOG 4599试验中的一项小型前瞻性附属试验^[129]^。研究发现，VEGF的基线血浆水平可预测PFS。另外，基线水平较高的患者对贝伐单抗联合化疗的有效率显著优于单独化疗，且低水平的VEGF对有效率或生存无预测价值。

2009版推荐D2。为了获得组织以达到更为精确的组织学分类或研究目的，更新委员会支持通过合理的努力以获得比常规细胞学标本更多的组织。

文献更新。传统上，不论肿瘤的组织学类型或分子亚型，Ⅳ期NSCLC患者均可选择化疗方案。而且，Ⅳ期NSCLC的诊断通常依赖细针抽取的细胞学标本，这些细胞学标本不足以为组织学分类或额外的细胞检测提供充足数量的细胞。当治疗选择受限时，该方法可行。然而，新近数据表明此种模式正在改变。对于特定组织学亚型，一些药物似乎更为有效或毒性较低（见指南全文中一线推荐A2、A5、A7及A8后的讨论）。而且，最近的研究表明基于分子标记物选择药物可能会潜在地提高疗效。整体上，这些趋势已促使人们基于最大获益与最低毒性的原则去采用最优治疗方案。

更新委员会亦已意识到参照组织学或分子分类纳入样本对在研以及筹备中的新药或药物联合的临床试验的重要性。分子检测有助于将NSCLC患者重新分为不同的亚组，从而在各亚组应用不同的最佳治疗方法。一些研究，如：为对厄洛替尼或吉非替尼产生获得性耐药的患者研发新药，报道了在临床过程中接受连续活检的患者中可观察到EGFR TKI治疗的获得性分子改变。

因此，尤其是在患者的常规治疗中，更新委员会支持为获取比常规细胞学标本更多的组织而进行的合理的努力。更新委员会意识到，对于患者的控制来说，区分鳞癌与非鳞癌NSCLC或获得可用于分子检测的额外组织的能力可能是很有价值的，但并不是必不可少的。例如，能排除NSCLC患者中鳞癌组织学类型的情况下可使肿瘤学家考虑应用可使患者死亡危险降低20%-30%的药物，这可能会使患者的中位生存时间延长大约2个月。当考虑使用创伤性更强或额外的活检时，如中心活检，主治肿瘤学家必须平衡基于选择药物的改善疗效的潜能与延误治疗设计到的和/或活检本身对患者个体的危险。

对于方法和研究的全部讨论，包括表 2列出的方法和研究，可参考指南的网络版，可登陆www.asco.org/guidelines/nsclc；也可从基于指南的临床工具与资源中获得。

## 未来研究方向

Ⅳ期NSCLC患者的生存现状仍不容乐观，在疾病治疗过程的任一时间，所有的合格患者均应被鼓励参与到临床研究试验中。更多有关改善医生与Ⅳ期NSCLC患者的沟通策略的研究可能会改善共同决策的制定，并提高研究的参与性。

更新委员会用于评价的现有数据促进了对老年患者和/或PS欠佳患者的特别关注。今后的研究需关注这些亚组。在今后的研究中，应以利用老年评估方法确定的生理年龄来定义老年人。PS≥2分的NSCLC患者应区别于受合并性疾病损害的患者。

最新的临床试验发现了其它一些具有预后和/或预测价值的肿瘤或患者特征，包括组织学类型（鳞癌*v*非鳞癌）^[27]^、分子亚组（*EGFR*突变、*EGFR*扩增、EGFR表达、或*KRAS*突变）^[58, 87, 91, 92, 104, 113]^、曾接受过的治疗数量（曾接受过0、1或≥2次治疗）^[72, 73]^、曾接受的治疗时间^[72]^、吸烟状态（包括从不吸烟者[终生 < 100支] ^[57, 74, 140]^、既往轻微吸烟者[ < 15包-年]、以及严重吸烟者[≥15包-年]）^[55, 74, 140]^以及亚裔^[58, 74]^。尽管在纳入中可能存在不平衡性，今后的临床试验应考虑以下方面：使患者获益最大，并根据包括PS、性别、吸烟状态、曾接受过的治疗以及分子特征等预后因素进行分层。在应用于前瞻性临床试验中之前，应优化并标准化用于肿瘤组织分子检测的工具。最理想的情况是，改进新技术，以使它们能更灵活地应用于社区医疗中。

## 患者-医生沟通

与指南推荐不同，该部分描述的一致性意见并非基于系统评价中的证据，而是基于利用问卷、调查、采访、办公室咨询观察和分析以及其它定性研究方法的文献。

NSCLC患者通常伴有复杂的医学、心理学及社会问题，在探讨治疗之前医生必须考虑这些问题。例如，肺癌患者会出现由于诊断为预后不良的NSCLC而情绪低落、丧失自我、惧怕疼痛和死亡和/或失落感。由于其与吸烟有关，肺癌具有特殊的社会恶名。一些患者很愤怒，因为他们从不吸烟却被强加上吸烟的这种社会耻辱。时间和培训的不足、系统水平的障碍和/或患者因素，如对疾病和治疗的误解，对医生的有效沟通构成障碍。

有关医生与NSCLC患者沟通的研究发现医患沟通间存在如下问题：错失表达同情的机会^[141]^、临床医生使用责备词语、缺乏预后讨论（20%的患者无意讨论预后信息）^[142]^，且不同种族/伦理背景下医生与患者间缺乏信息交流与信任。

尽管有效的沟通较难以实现，研究表明强化训练有助于医生与癌症患者间更有效地交流^[141, 144]^。提供一个仅限于治疗方案讨论的对话，不失为一种让患者参与到共同决策制定中的方法；医生应采用个性化描述的方式告知患者他或她的个体危险及获益。支持Ⅳ期NSCLC制定共同治疗决策的另一因素为监护者的作用，有助于临床肿瘤学家阐明有益的信息^[143, 145, 146]^。在治疗方案部分，获益与危险间的平衡很重要。肺癌患者乐于接受治疗（如化疗）的出发点差异悬殊^[147-149]^。医生们推断的NSCLC患者乐意接受治疗的想法可能并不准确，或可能与患者的态度表现不一致^[147]^。肺癌患者可能过高估计了潜在毒性治疗的生存获益^[150, 151]^。因此，评估患者的偏好以及他/她对Ⅳ期NSCLC化疗或生物治疗危险与获益的了解，对于医生来说是明智的^[150]^。整个指南对NSCLC治疗的获益与危险也进行了深入讨论，并建议医生考虑使用协商语气。

## 健康状况差异

指南的该部分基于对文献的系统审视。在肺癌中，种族与民族差异显著。对于肺癌的所有分期，与白种人相比，其它种族及少数民族的预后较差。肺癌公共卫生服务的差异归因于患者的危险行为（吸烟、烟雾吸入、以及每天吸烟的数目）、社会经济状态（包括受教育水平）、健康服务的水平、以及合并性疾病^[152-154]^。健康差异通常是由于公共卫生服务提供者与患者间的沟通不力。当患者接受相同的治疗时，种族组别间的预后差异最小化^[152]^。对接受公共卫生服务产生的这些差异的认识应在Ⅳ期NSCLC临床实践指南更新的背景下进行考虑，且公共卫生服务的提供者应为所有的患者提供最高水平的癌症治疗。

指南所讨论的药物的经济成本不同，而且尽管成本-效益分析不在指南范畴内且不会影响推荐，但表 3依然提供了基于医疗保险与医疗救助中心（Rochville, MD）的数据的粗略花费以供参考。

**3:** Ⅳ期NSCLC的治疗方案及费用（对于BSA为1.96的患者[体重=81.5 kg，身高=169 cm，来自2009年7月1日医疗保险计划B^*^的补偿]）

治疗模式	药物	初始剂量	HCPCS编码剂量（mg）	肠外治疗医疗保险支付限额（＄）	购买1份保险的肠外治疗1次的补偿或口服药物治疗1个月的花费（＄）	方案	两个周期的花费（＄）
一线	贝伐单抗†	15 mg/kg （仅建议与卡铂/紫杉醇联合）‡	10	57	7 020§	每三周	14 040
一线	卡铂†	700 mg， 每三周‡	50	5	73	每三周	146
一线	西妥昔单抗†	初始剂量为 400 mg/m^2^， 随后为250 mg/m^2^ （仅推荐与顺铂/长春瑞滨联合）‡	10	50	3 894§ （备注：仅初始剂量）	每周	18 981
一线	顺铂†	75 mg/m^2^， 每三周	50	12	34§	每三周	68║
一线及二线	多西紫杉醇†	75 mg/m^2^， 每三周	20	345	2 530§	每三周	5 060
二线	厄洛替尼¶	150 mg/d‡	——	——	4 557	每天	9 114
一线及二线	吉非替尼¶	250 mg/d‡	——	——	2 127	每天	4 255
一线	吉西他滨†	1 250 mg/m^2^‡	200	141	1 729§	第1、8天， 每三周	6 914
一线	依立替康†	60 mg/m^2^	20	15	88§	第1、8、15天， 每四周	527
一线	紫杉醇†	200 mg/m^2^	30	8	101§	每三周	201
一线及二线	培美曲塞†	500 mg/m^2^ （备注：在二线化疗中的单一疗法）‡	10	49	4 841§	每三周	9 682
一线	长春瑞滨†	25 mg/m^2^	10	13	64§	第1、8天， 每三周一次	257
备注：药物价格通过第三方付费者的观点进行评估，并基于被药物供给方广泛接受的医疗保险与医疗救助中心的补偿率，通过药品生产商的平均销售价格计算得出。未考虑其它治疗相关的直接和间接花费，如输液、止吐药以及诊断性实验检测或影像，如计算机断层扫描。实际的治疗花费及补偿因区域、患者、组织、实践以及时间而不同，读者应根据自身实际情况咨询当地费用信息。药物的商品名及生产商如下：贝伐单抗（Avastin; Genentech, South San Francisco, CA）、西妥昔单抗（Erbitux; Bristol-Myers Squibb, Princeton, NJ; ImClone Systems, New York, NY）、厄洛替尼（Tarceva; Genentech/Roche, South San Francisco, CA）、吉非替尼（Iressa; AstraZeneca, Wilmington, DE）、培美曲塞（Alimta; Eli Lilly, Indianapolis, IN）以及吉西他滨（Gemzar; Eli Lilly）。 简写：NSCLC：非小细胞肺癌；BSA：体表面积；HCPCS：普通健康处理编码系统。 *口服制剂厄洛替尼和吉非替尼除外。 †可注射药物的花费基于2009年7月1日生效的医疗保险B部分支付限额。（无其它用药费用及调整；医疗保险B部分平均售价见http://www.cms.hhs.gov/McrPartBDrugAvgSalesPrice/01a1_2009aspfles.asp）。 ‡药物剂量专门列于指南中。其它剂量基于大多数临床试验中的剂量。 §根据体重81.5 kg、身高169.25 cm的成年人计算得出。（McDowell MA, Fryar CD, Ogden CL, *et al*:儿童及成人权威参考数据: United States, 2003-2006. National Health Statistics Reports. Hyatsville, MD, National Center for Health Statistics, 2008）。男性≥20岁=平均体重，88.3 kg；平均身高，176.3 cm；女性≥ 20岁=平均体重，74.7 kg；平均身高，162.2 cm。Mosteller公式用以计算BSA）。 ║与水化成本显著相关。 ¶口服药物成本代表门诊医疗处方。计算如下：假定30天处方（无配药费），两个月花费加倍（Medscape: Erlotinib oral. http://www.medscape.com/druginfo/pricebrandimage?cid_med&drugid_92157&drugname_Erlotinib_Oral&monotype_pricebrandimage; Medscape: Gefitinib oral. http://www.medscape.com/druginfo/pricebrandimage?cid_med&drugid_75201&drugname_Geftinib_Oral&monotype_pricebrandimage）。 注：本表获得© 2009 by American Society of Clinical Oncology复制许可。							

## ASCO指南

ASCO实践指南基于当时已发表的临床证据和文献，反映了专家们的共识，旨在帮助医生制定临床决策，并提出今后研究的问题及思路。由于肿瘤学方面科学信息的急速发展，自指南提交至发表以来，新的证据可能已出现。指南并未持续更新，故可能未反映最新的进展。指南并不能解释患者间的个体差异，且不能认为指南纳入了所有适当的治疗方法或排除了其它治疗。依照对患者的独立经验与了解以决定患者最佳的治疗措施，是临床医生或其它公共卫生服务提供者的责任。因此，遵从何种指南均属自愿，医生应根据每一患者的具体情况而制定最终的治疗方案。ASCO指南描述了在临床实践中方法及治疗的使用，但不能在临床试验的背景下主观地使用这些干预措施。对起于或与ASCO指南使用相关的对患者或物品的损害或破坏，或任何错误或遗漏，ASCO均不承担责任。

## GUIDELINE AND CONFLICTS OF INTEREST

Te Update Commitee was assembled in accordance with ASCO’ s Conflict of Interest Management Procedures for Clinical Practice Guidelines. Membersof the panel completedASCO’s disclosure form, which requires disclosure of financial and other interests that are relevant to the subject mater of the guideline, including relationships with commercial entities that are reasonably likely to experience direct regulatory or commercial impact as the result of promulgation of the guideline. Categories for disclosure include employment relationships, consulting arrangements, stock ownership, honoraria, research funding, and expert testimony. In accordance with the Procedures, the majority of the members of the Commitee did not disclose any of these relationships. Disclosure information for each member of the Commitee is published adjunct to this guideline.

## DEDICATION

This guideline is dedicated to the memories of Karen Parles and Anita Johnston who, while living with NSCLC, educated, influenced, and inspired countless clinicians, researchers, advocates, and others with and affectedby NSCLC. Their work and their memories will continue to do so.

## AUTHORS’ DISCLOSURES OF POTENTIAL CONFLICTS OF INTEREST

Although all authors completed the disclosure declaration, the following author(s) indicated a financial or other interest that is relevant to the subject matter under consideration in this article. Certain relationships marked with a “U” are those for which no compensation was received; those relationships marked with a “C” were compensated. For a detailed description of the disclosure categories, or for more information about ASCO’s conflict of interest policy, please refer to the Author Disclosure Declaration and the Disclosures of Potential Conflicts of Interest section in Information for Contributors.

**Employment or Leadership Position: None Consultant or Advisory Role:** Julie Brahmer, Eli Lilly (C), ImClone Systems (C); Joan H. Schiller, Genentech (C), AstraZeneca (C), Pfizer (C), Eli Lilly (C) **Stock Ownership:** None **Honoraria:** Sherman Baker Jr, Lilly Oncology; Janessa L. Laskin, Hoffman-La Roche, Eli Lilly, AstraZeneca **Research Funding:** Christopher G. Azzoli, Allos Therapeutics, sanofi-aventis, Genentech BioOncology; Dav id H. Johnson, Merck; Janessa L. Laskin, HoffmanLa Roche; David G. Pfister, sanofi-aventis, Eli Lilly, AstraZeneca, Genentech; Joan H. Schiller, ArQuele, Pfizer, Merck **Expert Testimony:** None **Other Remuneration:** Willam Pao, Molecular MD

## AUTHOR CONTRIBUTIONS

**Conception and design:** Christopher G. Azzoli, Giuseppe Giaccone

**Administrative support:** Christopher G. Azzoli, Sarah Temin

**Collection and assembly of data:** Christopher G. Azzoli, Sarah Temin Data analysis and interpretation: Christopher G. Azzoli, Sherman Baker Jr, William Pao, David H. Johnson, David G. Pfister, Joan H. Schiller, Thomas J. Smith, Giuseppe Giaccone

**Manuscript writing:** Christopher G. Azzoli, Sherman Baker Jr, Sarah Temin, William Pao, David H. Johnson, David G. Pfster, Joan H. Schiller, Thomas J. Smith, John R. Strawn, Giuseppe Giaccone

**Final approval of manuscript:** Christopher G. Azzoli, Sherman Baker Jr, William Pao, Timothy Aliff, Julie Brahmer, David H. Johnson, Janessa L. Laskin, Gregory Masters, Daniel Milton, Luke Nordquist, David G. Pfister, Steven Piantadosi, Joan H. Schiller, Reily Smith, Thomas J. Smith, John R. Strawn, David Trent, Giuseppe Giaccone
